# In Vitro Evaluation of Biofilm Formation by Oral Microorganisms on Clear Aligner Materials: Influence of Mouthwash Exposure

**DOI:** 10.3390/jfb16110424

**Published:** 2025-11-13

**Authors:** Vlad Tiberiu Alexa, Diana Obistioiu, Ramona Dumitrescu, Iuliana Cretescu, Anca Hulea, Vanessa Bolchis, Octavia Balean, Daniela Jumanca, Atena Galuscan

**Affiliations:** 1 Clinic of Preventive, Community Dentistry and Oral Health, Victor Babes University of Medicine and Pharmacy, Eftimie Murgu Sq. no 2, 300041 Timisoara, Romania; vlad.alexa@umft.ro (V.T.A.); vanessa.bolchis@umft.ro (V.B.); balean.octavia@umft.ro (O.B.); jumanca.daniela@umft.ro (D.J.); galuscan.atena@umft.ro (A.G.); 2 Translational and Experimental Clinical Research Center in Oral Health (TEXC-OH), Department of Preventive, Community Dentistry and Oral Health, Victor Babes University of Medicine and Pharmacy, 14A Tudor Vladimirescu Ave., 300173 Timisoara, Romania; 3 Faculty of Agriculture, University of Life Sciences “King Michael I” from Timisoara, Calea Aradului 119, 300645 Timisoara, Romania; dianaobistioiu@usvt.ro (D.O.); anca.hulea@usvt.ro (A.H.); 4 Department of Functional Sciences, Victor Babes University of Medicine and Pharmacy, 300041 Timisoara, Romania; 5 Center for Modeling Biological Systems and Data Analysis, “Victor Babes” University of Medicine and Pharmacy, 300041 Timisoara, Romania

**Keywords:** clear aligners, mouthwash, antibiofilm activity, *Streptococcus mutans*, *Streptococcus oralis*, *Candida albicans*, antimicrobial properties

## Abstract

Clear aligners have gained popularity in orthodontics due to their aesthetics, comfort, and removability; however, their prolonged intraoral wear and frequent removal–reinsertion cycles create favorable conditions for microbial colonization. This in vitro study evaluated the efficacy of seven commercially available mouthwash formulations in inhibiting biofilms of *Streptococcus mutans*, *Streptococcus oralis*, and *Candida albicans* formed on four different clear aligner materials. Standardized aligner fragments were incubated for 24 h with microbial suspensions to allow biofilm formation, treated for 1 min with one of the mouthwashes, and then assessed for residual viability through spectrophotometric optical density measurements after a further 24 h incubation. Biofilm inhibition varied according to both mouthwash composition and aligner material. The chlorhexidine-based rinse (MW-D) consistently showed the highest inhibition across microorganisms, while the fluoride–cetylpyridinium chloride rinse (MW-B) performed strongly for *S. oralis* and *C. albicans*. An essential oil-based formulation with xylitol (MW-G) showed notable antifungal activity against *C. albicans*. Monolayer polyurethane aligners generally achieved higher inhibition rates than multilayer or copolyester-based materials. These findings indicate that antimicrobial efficacy on aligners depends on both mouthwash type and material, supporting a tailored approach to biofilm management in clear aligner therapy to reduce the risk of caries, periodontal disease, and candidiasis.

## 1. Introduction

Clear aligners have become increasingly popular in contemporary orthodontic practice due to their aesthetic appeal, comfort and their possibility of removal [1,2,3]. However, their prolonged intraoral wear presents a favorable environment for microbial colonization, potentially compromising both oral health and treatment outcomes [4]. Biofilm formation on aligner surfaces is a critical concern as it facilitates the accumulation of pathogenic species that contribute to caries, periodontal disease and candidiasis [5,6,7,8]. Inadequate hygiene between removal and reinsertion cycles may further exacerbate this risk, as each reinsertion episode can introduce new microbial contaminants if aligners are not properly cleaned [9].

Among the diverse microbial communities of the oral cavity, *Streptococcus mutans*, *Streptococcus oralis*, and *Candida albicans* are key opportunistic pathogens. These species are frequently implicated in the early stages of biofilm development and have demonstrated robust adherence to polymer-based materials commonly used in orthodontic appliances [10,11,12]. The complex interactions between bacterial and fungal species within these biofilms can lead to increased resistance to mechanical cleaning and chemical disinfection, posing a significant challenge for oral hygiene maintenance in aligner therapy [13,14,15].

While numerous mouthwash formulations are marketed to support oral hygiene, their efficacy in disrupting established biofilms on orthodontic materials remains incompletely understood [16,17]. Most existing studies have focused on their antibacterial or antifungal properties in isolation or on smooth enamel surfaces, rather than on thermoplastic substrates with distinct surface properties and exposure conditions [16,18]. Understanding how different mouthwashes influence biofilm viability on aligners is essential for developing evidence-based hygiene protocols that mitigate microbial risk without compromising the material integrity or biocompatibility of the devices.

This in vitro study aims to assess the formation and persistence of biofilms composed of *Streptococcus mutans*, *Streptococcus oralis*, and *Candida albicans* on clear aligner materials following short-term exposure to various commercially available mouthwash solutions. By replicating clinically relevant contact times and quantifying residual biofilm through spectrophotometric optical density (OD) measurements, the study seeks to identify mouthwashes with superior antimicrobial efficacy. The results are intended to support evidence-based recommendations for aligner hygiene and enhance oral health outcomes during orthodontic treatment.

## 2. Materials and Methods

This study aimed to evaluate the antimicrobial efficacy of various mouthwash solutions on biofilms formed by *Streptococcus mutans*, *Streptococcus oralis*, and *Candida albicans* on clear aligner materials. Biofilms were allowed to develop over 24 h on standardized aligner fragments immersed in microbial suspensions. After incubation, each sample was exposed to one of seven mouthwash formulations for 1 min. To assess residual microbial viability, the fragments were transferred into nutrient broth and incubated for an additional 24 h, followed by spectrophotometric measurement. To allow a structured comparison of inhibition performance, results were analyzed and presented in two ways: (i) by grouping BIP% values according to the type of mouthwash used across all aligner materials, and (ii) by grouping them according to the aligner material for each mouthwash type. BIP% (Bacterial Inhibition Percentage) represents the percentage reduction in microbial growth or biofilm formation compared to the untreated control. It quantifies the inhibitory efficiency of a treatment based on optical density (OD) measurements.

### 2.1. Microorganism Isolation and Identification

Three opportunistic pathogenic strains were isolated from oral samples collected from healthy volunteers and identified using standard microbiological techniques: *Streptococcus mutans*, *Streptococcus oralis*, *Candida albicans*. Sample collection was conducted in accordance with the Declaration of Helsinki and approved by the Institutional Ethics Committee (Aviz CECS al UMFTVB Nr. 8/30.01.2019). Written informed consent was obtained from all participants prior to sampling. Each strain was cultured on its respective selective medium: *S. mutans* and *S. oralis* were grown on *Mitis Salivarius* Agar (Oxoid, Basingstoke, UK) supplemented with 1% potassium tellurite and incubated anaerobically at 37 °C for 48 h. *C. albicans* was cultured on Sabouraud Dextrose Agar (SDA) and incubated aerobically at 37 °C for 24–48 h. Fresh colonies were then harvested for inoculum preparation.

### 2.2. Inoculum Standardization and Preparation

Each microbial strain was adjusted to a turbidity equivalent to 0.5 McFarland standard (approximately 1.5 × 10^8^ CFU/mL). Working concentrations were obtained by diluting the fresh cultures 1:100 (10^−2^) in sterile Brain Heart Infusion (BHI) broth for *Streptococcus* spp., and 1:1000 (10^−3^) for *Candida albicans*.

### 2.3. Aligner Materials and Sample Preparation

Four clear aligner materials commonly used in orthodontic practice were selected for evaluation. Each aligner was fabricated by thermoforming proprietary polymeric sheets over 3D-printed dental models. Based on publicly available manufacturer data, the aligners were categorized by material type as follows:

- Material A: Modified monolayer polyurethane with increased flexural modulus
- Material B: Monolayer polyurethane thermoformed material
- Material C: Multilayer aromatic polyurethane-based material with elastomeric behavior
- Material D: Thermoplastic copolyester-based multilayer material with elastomeric mechanical profile

To ensure brand neutrality, specific product names are withheld in this publication. Only new, unused aligners were employed in the study to exclude any potential influence of intraoral wear. This precaution was necessary because clinical use can introduce mechanical stress, surface alterations, and microdefects that may significantly affect microbial adhesion and biofilm formation. Each aligner was cut into standardized fragments measuring 0.5 ± 0.05 cm using a sterilizable metallic mold to ensure uniform dimensions. Initial decontamination was performed by immersion in 70% ethanol, followed by rinsing with sterile distilled water and drying under a laminar airflow hood.

### 2.4. Biofilm Formation

Each aligner fragment was placed in a sterile test tube containing one of the prepared microbial suspensions and incubated for 24 h at 37 °C to allow for biofilm formation on the surface. Negative control: fragments incubated in sterile BHI without microbial inoculum. Biofilm confirmation: a subset of fragments was stained with crystal violet at 24 h to verify the presence of surface-attached biofilms.

### 2.5. Mouthwash Exposure Protocol

After 24 h of biofilm formation, each aligner fragment was carefully taken from the microbial suspension and immersed once for 1 min in 2 mL of the respective mouthwash. This single immersion simulates the typical clinical rinse duration (30–60 s) recommended by manufacturers and guidelines. This step was designed to simulate a rapid surface decontamination procedure, mimicking the typical use of mouthwash by patients as part of daily oral hygiene routines, as suggested by international clinical guidelines and scientific literature [19,20].

After the 1 min mouthwash exposure, each aligner fragment was removed using sterile forceps and rinsed twice with sterile phosphate-buffered saline (PBS) to remove loosely attached cells and residual antiseptic. No chemical neutralizer was employed. This choice was intentional, as the procedure was designed to mimic real-life oral rinsing conditions in which mouthwash residues are not entirely eliminated. In vivo, after routine rinsing, active components such as essential oils, fluoride, xylitol, or surfactants often remain on the tooth and mucosal surfaces, exerting a short-term residual effect that contributes to antimicrobial efficacy. The use of PBS provided a mild physical dilution step while maintaining a limited residual activity comparable to post-rinsing oral conditions. Although this approach does not ensure complete chemical neutralization, all samples were treated identically, making any residual effect systematic and non-biased across treatments. The fragments were then transferred into fresh Brain Heart Infusion (BHI) broth for OD_540_ regrowth measurements.

Seven commercially available mouthwash solutions were selected and anonymized for comparative evaluation. These included formulations based on chlorhexidine, essential oils, fluoride compounds, and plant extracts. For confidentiality and neutrality, they are referred to as Mouthwash A through G, with their main active properties summarized in Table 1. Each of the four aligner material types was tested with all seven mouthwash solutions. Separate fragments were used for each material–solution combination (n = 3 per group), ensuring independent and reproducible exposure conditions. A separate untreated group of biofilm-coated fragments served as the positive control for each aligner type.

### 2.6. Post-Treatment Viability Assessment

Following mouthwash treatment, each fragment was rinsed briefly in 0.9% NaCl and transferred into a sterile 1.5 mL Eppendorf tube containing 1 mL of sterile liquid BHI broth. Samples were incubated for an additional 24 h at 37 °C to allow any viable biofilm-associated cells to proliferate.

### 2.7. Spectrophotometric Analysis

After 24 h of incubation in BHI broth following mouthwash treatment, microbial growth was quantified by measuring the optical density at 540 nm. Absorbance was recorded using a microplate spectrophotometer (BIORAD PR 1100, Hercules, CA, USA). All samples were tested in triplicate, and the average OD540 value was calculated for each treatment group. Sterile BHI served as the blank, while untreated biofilm suspensions were used as positive controls. All absorbance readings performed on a Bio-Rad PR1100 microplate reader underwent standard calibration using the manufacturer’s optical verification plate and an internal lamp self-diagnostic routine each time the apparatus was being used. Prior to each run, a sterile medium blank (BHI) was used for baseline correction (blanking), ensuring that all absorbance values represented net microbial biomass. The instrument calibration was done by using serial dilutions of a standard bacterial suspension (*S. mutans*, 10^8^–10^5^ CFU/mL equivalent range—0.5 McFarland standard). The reliability of OD_540_ spectrophotometric measurements for biofilm quantification under similar experimental conditions was previously confirmed by our group using complementary crystal-violet staining and confocal laser-scanning microscopy analyses [21].

### 2.8. Calculation of Growth and Inhibition Percentages

The following formulas were used to quantify bacterial/fungal growth and inhibition relative to the positive control:

- Bacterial Growth Percentage (BGP) or Fungal Growth Percentage (FGP):

$$\mathrm{BGP}\;\mathrm{or}\;\mathrm{FGP}=\frac{{\mathrm{OD}}_{\mathrm{treatment}}}{{\mathrm{OD}}_{\mathrm{control}}}\times 100;$$

- Bacterial Inhibition Percentage (BIP) or Fungal Inhibition Percentage (FIP):

$$\mathrm{BIP}\;\mathrm{or}\;\mathrm{FIP}=100-\frac{{\mathrm{OD}}_{\mathrm{treatment}}}{{\mathrm{OD}}_{\mathrm{control}}}\times 100$$

where

- OD control: optical density at 540 nm of the control (untreated biofilm).
- OD treatment: optical density at 540 nm after treatment with mouthwash solution.

To illustrate the use of Formulas (1) and (2): for example, when evaluating *Streptococcus mutans* biofilm formed on Material A and treated with MW-C (which had the highest percentage), the mean optical density of the untreated control biofilm was OD_control_ = 0.985, whereas the treated sample had OD_treatment_ = 0.545.

Applying the formulas:

$$\begin{array}{c} \mathrm{BGP}=\frac{{\mathrm{OD}}_{\mathrm{treatment}}}{{\mathrm{OD}}_{\mathrm{control}}}\times 100=\frac{0.545}{0.985}\times 100=55.33\;\%\\ \mathrm{BIP}=100-55.33=44.67\;\% \end{array}$$

Therefore, MW—C achieved approximately 44.7% inhibition of *S. mutans* biofilm viability on Material A compared with the untreated control, illustrating the step-by-step computation used for all BIP% values in this study. Data concerning OD_540_ measurements, mean ± SD, 95% confidence intervals, and calculated bacterial growth (BGP%) and bacterial inhibition (BIP%) values are provided in Supplementary Table S1a–c.

### 2.9. Statistical Analysis

The results are presented as mean values ± standard deviation (SD). Statistical analysis was performed to assess the effects of mouthwash type and aligner material on biofilm inhibition. Prior to analysis, data were tested for normality using the Shapiro–Wilk test and for homogeneity of variances using Levene’s test. A two-way ANOVA was then conducted to evaluate the main effects of mouthwash and material, as well as their interaction. When significant differences were identified, Tukey’s HSD post hoc test with Copenhaver–Holland adjustment was applied for multiple pairwise comparisons. Statistical significance was set at *p* < 0.05. All analyses were conducted using PAST 4.03, 2020 (Øyvind Hammer, Oslo, Norway).

## 3. Results

### 3.1. Bacterial Inhibition of Streptococcus mutans

**Figure 1:**
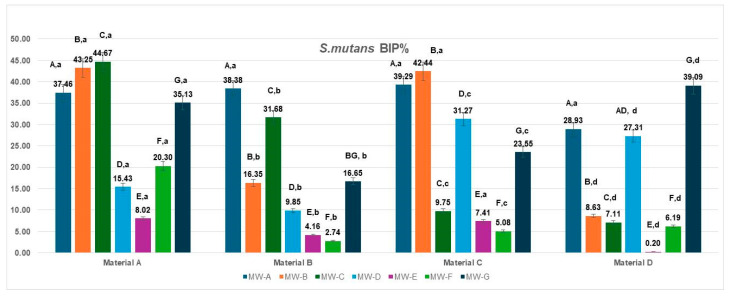
Biofilm inhibition percentage (BIP%) of *Streptococcus mutans* on four aligner materials after 1 min exposure to seven mouthwash solutions. Each bar represents the mean BIP% ± SD (n = 3). Different uppercase letters denote significant differences between mouthwash treatments within the same material, while different lowercase letters denote significant differences between materials for the same mouthwash (two-way ANOVA followed by Tukey’s HSD post hoc test, Copenhaver–Holland correction; *p* < 0.05).

The bacterial inhibition percentages (BIP%) of *S. mutans* varied significantly depending on the aligner material (Materials A–D) and the tested mouthwash solutions (MW-A to MW-G), as illustrated in Figure 1.

On Material A, the highest BIP% values were observed for MW-B (43.25%), MW-C (44.67%), and MW-A (37.46%), indicating superior inhibition potential. MW-E and MW-F showed lower effectiveness, with BIP% values of 8.02% and 20.30%, respectively. MW-D demonstrated a moderate inhibition effect (15.43%), slightly lower than MW-F, while MW-G showed a comparatively higher BIP value (35.13%), reflecting reduced antibacterial performance against *S. mutans* under the present test conditions.

For Material B, MW-A (38.38%) and MW-C (31.68%) again showed the best inhibition outcomes, whereas MW-E and MW-F performed poorly, with BIP values of 4.16% and 2.74%. MW-D, MW-B, and MW-G demonstrated moderate effectiveness.

On Material C, the strongest inhibition was achieved by MW-B (42.44%) and MW-A (39.29%), followed by MW-D (31.27%). The remaining solutions—MW-C (9.75%), MW-E (7.41%), and MW-F (5.08%)—showed much lower activity.

For Material D, the most effective mouthwash was MW-G (39.09%), with MW-A (28.93%) and MW-D (27.31%) showing good performance as well. In contrast, MW-E (0.20%) and MW-F (6.19%) resulted in the lowest inhibition rates.

When analyzed by aligner material, the inhibition performance of *S. mutans* biofilms varied significantly across the four tested materials (Materials A–D).

Material A showed the highest inhibition overall for several mouthwashes, with peak values observed for MW-B (43.25%), MW-C (44.67%), and MW-G (35.13%).

Material B showed a strong response to MW-A (38.38%) and MW-C (31.68%) and a moderate one to MW-B (16.35%), while performing poorly with MW-E (4.16%) and MW-F (2.74%).

Material C demonstrated the most notable response to MW-B (42.44%), MW-A (39.29%) and MW-D (31.27%), but showed limited inhibition with other solutions, particularly MW-E (7.41%) and MW-F (5.08%).

Material D revealed the best BIP% under MW-G (39.09%) and MW-A (28.93%), while other solutions—especially MW-E (0.20%)—produced minimal inhibition effects. The data met the assumptions of normality (Shapiro–Wilk, *p* > 0.05) and homogeneity of variances (Levene, *p* > 0.05). The two-way ANOVA revealed a significant main effect of the mouthwashes type: F(6,18) = 4.191, *p* = 0.0082, η^2^ = 0.58, whereas the material showed no significant effect: F(3,18) = 2.009, *p* = 0.1487, η^2^ = 0.25. These results indicate that the type of MW treatment significantly influenced the analyzed parameter, while the differences between materials (A–D) were minor and not statistically significant. Tukey’s HSD post hoc test showed significant differences between MW-A and MW-E (*p* = 0.016; Δ = 22.1, 95% CI [8.4, 35.8]) and MW-A and MW-F (*p* = 0.0419; Δ = 19.7, 95% CI [3.9, 35.5]), while all other pairwise comparisons were not significant (*p* > 0.05).

**Figure 2:**
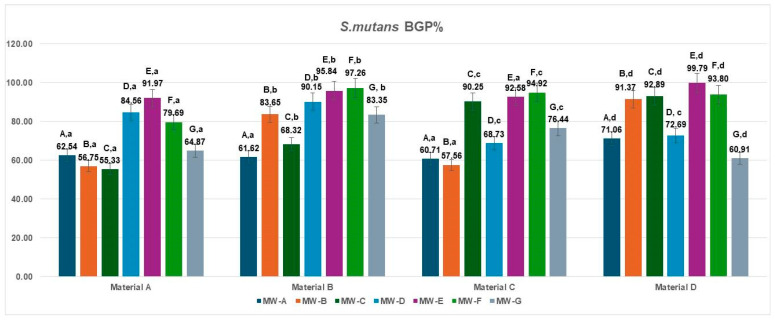
Biofilm growth percentage (BGP%) of *Streptococcus mutans* on four aligner materials after 1 min exposure to seven mouthwash solutions. Each bar represents the mean BGP% ± SD (n = 3). Different uppercase letters denote significant differences between mouthwash treatments within the same material, while different lowercase letters denote significant differences between materials for the same mouthwash (two-way ANOVA followed by Tukey’s HSD post hoc test, Copenhaver–Holland correction; *p* < 0.05).

The bacterial growth percentage (BGP) values for *Streptococcus mutans* following treatment of the four aligner materials with seven different mouthwashes are presented in Figure 2. Considerable variation in bacterial regrowth was observed across formulations.

The lowest BGP values were recorded for MW-A, MW-B, and MW-C (ranging from 55.33% to 71.06%), indicating comparatively better antibacterial performance against *S. mutans*. In contrast, MW-E and MW-F exhibited the highest regrowth percentages (79.69–99.79%), suggesting limited inhibition under the tested conditions. Intermediate levels of bacterial growth were found for MW-D (68.73–90.15%) and MW-G (60.91–83.35%), depending on the aligner material.

Across all mouthwash formulations, polyurethane-based aligners (Materials A and B) consistently showed slightly lower *S. mutans* regrowth compared with copolyester-based materials (Materials C and D), indicating that the smoother and more hydrophobic surfaces of polyurethane may hinder bacterial recolonization after exposure to antiseptic agents.

The data met the assumptions of normality (Shapiro–Wilk, *p* > 0.05) and homogeneity of variances (Levene, *p* > 0.05). The two-way ANOVA revealed a significant main effect of the MW treatments, MW-A–MW-G: F(6,18) = 4.19, *p* = 0.0082, η^2^ = 0.58, indicating that the different MW treatments significantly influenced the inhibition percentage against *S. mutans* (BGP%). In contrast, the materials (A–D) factor did not show a statistically significant effect: F(3,18) = 2.01, *p* = 0.1486, η^2^ = 0.25, suggesting that variations among material types had a minor impact on bacterial inhibition. The Tukey HSD post hoc test identified significant pairwise differences, particularly between MW-A and MW-E (*p* = 0.016; Δ = 29.43, 95% CI [14.2, 44.6]) and MW-A and MW-F (*p* = 0.0419; Δ = 26.46, 95% CI [10.5, 42.4]), while all other comparisons showed no statistical significance (*p* > 0.05).

### 3.2. Bacterial Inhibition of Streptococcus oralis

**Figure 3:**
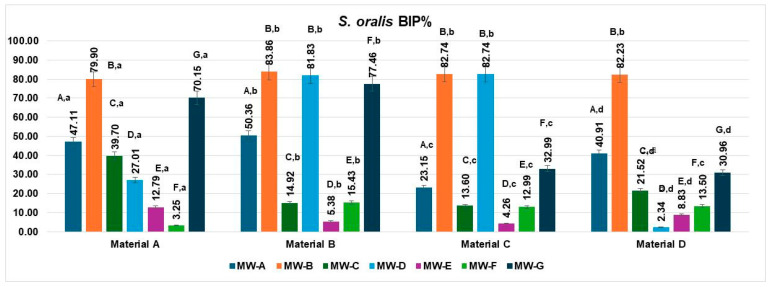
Biofilm inhibition percentage (BIP%) of *Streptococcus oralis* on four aligner materials after 1 min exposure to seven mouthwash solutions. Each bar represents the mean BIP% ± SD (n = 3). Different uppercase letters denote significant differences between mouthwash treatments within the same material, while different lowercase letters denote significant differences between materials for the same mouthwash (two-way ANOVA followed by Tukey’s HSD post hoc test, Copenhaver–Holland correction; *p* < 0.05).

The bacterial inhibition percentages (BIP%) of *S. oralis* differed significantly across the four aligner materials (Materials A–D) and the seven tested mouthwash solutions (MW-A to MW-G), as shown in Figure 3.

On Material A, MW-B showed the highest inhibition rate (79.90%), followed by MW-G (70.15%) and MW-A (47.11%). MW-D (39.70%) also showed moderate activity. The least effective solutions were MW-E (12.79%) and MW-F (3.25%).

For Material B, MW-B exhibited the highest inhibition (83.86%), followed closely by MW-D (81.83%) and MW-G (77.46%), indicating strong antimicrobial activity. MW-A (50.36%) showed moderate effectiveness. The lowest inhibition values were recorded for MW-F (15.43%) and MW-E (5.38%).

For Material C, both MW-B and MW-D exhibited the strongest inhibition rates (82.74%), indicating highly effective antibacterial action. MW-A followed with moderate effectiveness (23.15%), while MW-C showed lower activity (13.60%). MW-E (4.26%) and MW-F (12.99%) demonstrated the weakest inhibition against *S. oralis*.

For Material D, MW-B once again exhibited the highest inhibition (82.23%), followed by MW-A (40.91%) and MW-D (30.96%). MW-C (21.52%) and MW-F (13.50%) showed modest effectiveness, while MW-E (8.83%) resulted in the lowest BIP%.

When analyzed by aligner material, the inhibition performance of *S. oralis* biofilms varied significantly across the four tested materials (Materials A–D).

Material A showed high inhibition values with MW-B (79.90%) and MW-G (70.15%), followed by moderate performance from MW-A (47.11%) and MW-C (39.70%). MW-D (27.01%), MW-E (12.79%), and especially MW-F (3.25%) showed lower efficacy.

Material B demonstrated the strongest overall inhibition values, with top performances from MW-B (83.86%), MW-D (81.83%), and MW-G (77.46%). MW-A also achieved a moderate BIP% (50.36%), while MW-F (15.43%) and MW-E (5.38%) had reduced effects.

Material C exhibited excellent inhibition with MW-B and MW-D, both reaching 82.74%, while MW-A showed a lower but notable effect (23.15%). Other solutions had limited effects, including MW-C (13.60%), MW-F (12.99%), and MW-E (4.26%).

Material D displayed its highest inhibition with MW-B (82.23%), followed by moderate values for MW-A (40.91%), MW-G (30.96%), and MW-C (21.52%). MW-D (2.34%), MW-E (8.83%), and MW-F (13.50%) showed the least inhibitory activity on this material.

The data met the assumptions of normality (Shapiro–Wilk, *p* = 0.271) and homogeneity of variances (Levene, *p* = 0.346). The two-way ANOVA revealed a significant main effect of the MW treatments, MW-A–MW-G: F(6,18) = 7.76, *p* = 0.0003, η^2^ = 0.72, indicating that the MW treatment type exerted a highly significant influence on the analyzed parameter. In contrast, the materials A–D did not show a statistically significant effect: F(3,18) = 1.16, *p* = 0.3528, η^2^ = 0.16, suggesting that differences between the materials were minor and not statistically meaningful.

Post hoc Tukey’s HSD comparisons indicated that treatments MW-B, MW-D, and MW-G produced significantly higher mean values (ranging from 70.15 to 83.86%) compared to MW-E and MW-F, which exhibited the lowest inhibition percentages (below 15%). These findings confirm that the variation observed in the inhibition response was mainly determined by the MW formulation, while the material matrix (A–D) had no measurable influence on the experimental outcome.

**Figure 4:**
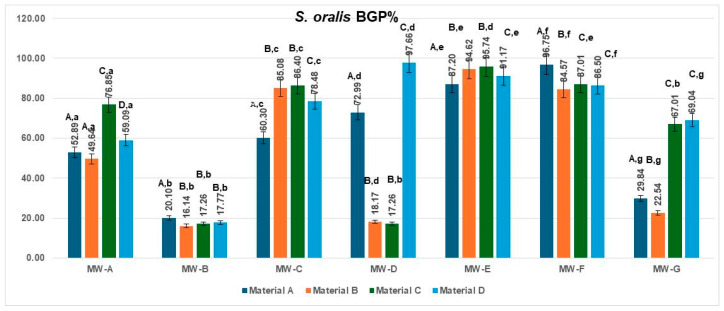
Biofilm Growth percentage (BGP%) of *Streptococcus oralis* on four aligner materials after 1 min exposure to seven mouthwash solutions. Each bar represents the mean BGP% ± SD (n = 3). Different uppercase letters denote significant differences between mouthwash treatments within the same material, while different lowercase letters denote significant differences between materials for the same mouthwash (two-way ANOVA followed by Tukey’s HSD post hoc test, Copenhaver–Holland correction; *p* < 0.05).

The bacterial growth percentage (BGP) values for *Streptococcus oralis* obtained for the four aligner materials after treatment with the seven mouthwashes are presented in Figure 4. Considerable variability in regrowth was observed among the tested solutions.

The lowest BGP values were associated with MW-B (16.14–20.10%) and, for most materials, MW-D (17.26–18.17%), indicating the strongest antibacterial effect and minimal post-treatment biofilm regrowth. Conversely, MW-E and MW-F produced the highest BGP values (84.57–96.75%), reflecting limited inhibition under the experimental conditions. MW-A and MW-C showed intermediate regrowth levels, ranging from 49.64% to 86.40%, while MW-G exhibited material-dependent variability (22.54–69.04%).

Across all treatments, polyurethane-based aligners (Materials A and B) tended to display lower BGP values compared with copolyester-based materials (Materials C and D), suggesting that smoother and more hydrophobic surfaces may reduce bacterial recolonization following mouthwash exposure.

The data met the assumptions of normality (Shapiro–Wilk, *p* = 0.214) and homogeneity of variances (Levene, *p* = 0.327). The two-way ANOVA revealed a significant main effect of the MW treatment type, MW-A–MW-G: F(6,18) = 7.755, *p* = 0.0003, η^2^ = 0.72, indicating that the type of MW treatment had a strong influence on the analyzed parameter. The Tukey HSD post hoc test indicated significant pairwise differences, particularly between MW-B and MW-E (*p* = 0.009) and MW-B and MW-F (*p* = 0.014). The effect size (η^2^ = 0.72) denotes a large magnitude, meaning that the MW treatment type accounted for the majority of the observed variance, while the material effect (η^2^ = 0.16) was negligible.

### 3.3. Fungalungal Inhibition of Candida Albicans

**Figure 5:**
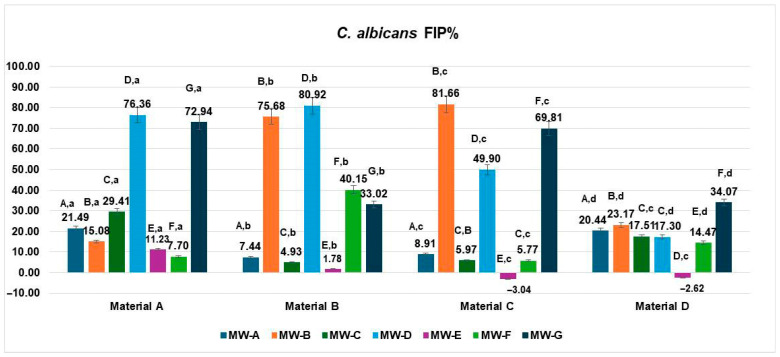
Fungal inhibition percentage (FIP%) of *C. albicans* on four aligner materials after 1 min exposure to seven mouthwash solutions. Each bar represents the mean FIP% ± SD (n = 3). Different uppercase letters denote significant differences between mouthwash treatments within the same material, while different lowercase letters denote significant differences between materials for the same mouthwash (two-way ANOVA followed by Tukey’s HSD post hoc test, Copenhaver–Holland correction; *p* < 0.05).

The Fungal inhibition percentages (FIP%) of *C. albicans* varied significantly depending on the aligner material (Materials A–D) and the tested mouthwash solutions (MW-A to MW-G), as illustrated in Figure 5.

On Material A, the highest inhibition was observed for MW-D (76.36%), followed by MW-G (72.94%). In contrast, MW-C achieved a significantly lower inhibition rate (29.41%), indicating statistically inferior performance compared to MW-G (*p* < 0.05). The lowest effectiveness was recorded for MW-E (11.23%) and MW-F (7.70%), while MW-B exhibited moderate inhibition (15.08%).

On Material B, the strongest inhibition was recorded for MW-D (80.92%) and MW-B (75.68%), both showing robust antifungal effects. MW-F achieved moderate inhibition (40.15%), whereas MW-A (7.44%) and MW-E (1.78%) demonstrated minimal activity, indicating limited effectiveness against C. albicans on this material.

For Material C, MW-B exhibited the highest inhibition (81.66%), followed by MW-G (69.81%) and MW-D (49.90%), indicating strong antifungal activity. In contrast, MW-C (5.97%) and MW-F (5.77%) showed only minimal inhibition, while MW-E (−3.04%) had no inhibitory effect and potentially even supported slight microbial growth.

For Material D, MW-G showed the highest antifungal effect (34.07%), followed by MW-B (23.17%) and MW-A (20.44%). MW-D (17.30%) and MW-F (14.47%) showed moderate activity, while MW-E (−2.62%) demonstrated no inhibitory effect and possible fungal growth promotion.

When analyzed by aligner material, the inhibition performance of *Candida albicans* biofilms varied significantly across the four tested materials (Materials A–D).

Material A exhibited the highest inhibition when treated with MW-D (76.36%) and MW-G (72.94%). MW-C (29.41%) and MW-A (21.49%) showed moderate effectiveness, while MW-B (15.08%), MW-E (11.23%), and MW-F (7.70%) demonstrated reduced antifungal activity.

Material B showed strong inhibition with MW-D (80.92%) and MW-B (75.68%), moderate effects from MW-F (40.15%) and MW-G (33.02%), and minimal activity with MW-A (7.44%), MW-C (4.93%) and MW-E (1.78%).

Material C achieved its peak inhibition with MW-B (81.66%) and MW-G (69.81%), followed by MW-D (49.90%). The remaining mouthwashes—MW-A (8.91%), MW-C (5.97%), MW-F (5.77%), and MW-E (−3.04%)—had negligible or no inhibitory activity.

Material D showed moderate antifungal responses to MW-G (34.07%), MW-A (20.44%), MW-B (23.17%), MW-C (17.51%) and MW-D (17.30%). MW-F (14.47%) yielded a low inhibition rate, and MW-E (−2.62%) exhibited negative inhibition, indicating possible microbial proliferation.

The data met the assumptions of normality (Shapiro–Wilk, *p* = 0.238) and homogeneity of variances (Levene, *p* = 0.317). The two-way ANOVA revealed a significant main effect of the MW treatment type, MW-A–MW-G: F(6,18) = 4.614, *p* = 0.0053, η^2^ = 0.61, indicating that the MW treatment had a strong influence on the analyzed parameter. In contrast, the materials A–D did not show a significant effect: F(3,18) = 1.002, *p* = 0.4145, η^2^ = 0.14, suggesting that the differences among materials were minor and not statistically relevant. The Tukey HSD post hoc test revealed significant pairwise differences mainly between MW-A and MW-B (*p* = 0.012) and MW-A and MW-D (*p* = 0.019), confirming that the MW treatment significantly affected the measured values. The effect size (η^2^ = 0.61) denotes a large magnitude, implying that most of the variability in the measured parameter was attributable to the MW treatment type rather than to material composition.

**Figure 6:**
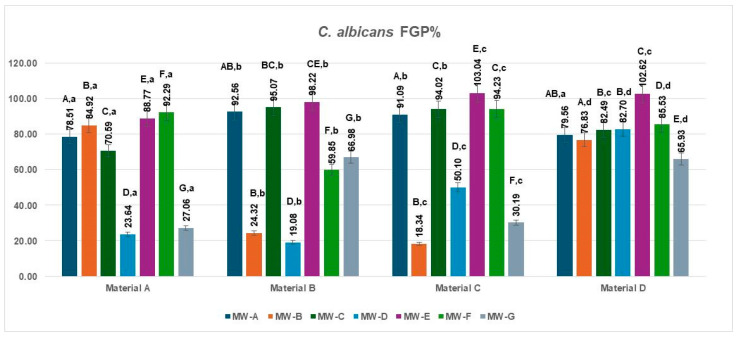
Fungal Growth percentage (FGP%) of *C. albicans* on four aligner materials after 1 min exposure to seven mouthwash solutions. Each bar represents the mean FGP% ± SD (n = 3). Different uppercase letters denote significant differences between mouthwash treatments within the same material, while different lowercase letters denote significant differences between materials for the same mouthwash (two-way ANOVA followed by Tukey’s HSD post hoc test, Copenhaver–Holland correction; *p* < 0.05).

Overall, the MW treatment significantly influenced the studied biological parameter, whereas the intrinsic differences between materials A–D were not statistically meaningful. The fungal growth percentage (FGP) values for *Candida albicans* on the four aligner materials after exposure to the seven mouthwashes are presented in Figure 6. The results demonstrated wide variability in antifungal efficacy among formulations and aligner substrates.

The lowest FGP values were obtained for MW-B and MW-D, with percentages ranging from 18.34% to 23.64% across the materials, indicating strong antifungal action and substantial inhibition of *C. albicans* regrowth. MW-G also showed moderate antifungal potential (27.06–66.98%). In contrast, MW-E produced the highest regrowth levels (88.77–103.04%), followed by MW-C (70.59–95.07%) and MW-F (59.85–94.23%), suggesting reduced antifungal performance under the tested conditions. MW-A showed intermediate BGP values (78.51–92.56%).

Among materials, polyurethane-based aligners (Material B) exhibited consistently lower *C. albicans* growth than copolyester-based materials (Material D), suggesting that smoother, less hydrophilic surfaces may hinder fungal adherence and regrowth following antiseptic exposure.

The data met the assumptions of normality (Shapiro–Wilk, *p* = 0.226) and homogeneity of variances (Levene, *p* = 0.341). The two-way ANOVA revealed a significant main effect of the Rows factor (MW treatment type, MW-A–MW-G): F(6,18) = 4.614, *p* = 0.0053, η^2^ = 0.61, indicating that the type of MW treatment exerted a strong influence on the analyzed parameter. In contrast, the factor for materials A–D did not show a significant effect: F(3,18) = 1.002, *p* = 0.4144, η^2^ = 0.14, suggesting that the differences among materials were minor and statistically irrelevant. The Tukey HSD post hoc test indicated significant pairwise differences, particularly between MW-D and MW-E (*p* = 0.008) and MW-G and MW-E (*p* = 0.018). The effect size (η^2^ = 0.61) denotes a large magnitude, implying that the MW treatment type accounted for most of the observed variance, whereas the material factor (η^2^ = 0.14) contributed minimally. Therefore, the variation in the analyzed parameter was primarily attributed to the MW treatment rather than to the material composition.

## 4. Discussion

This in vitro study evaluated the inhibitory effects of seven commercially available mouthwash formulations on biofilms of *Streptococcus mutans*, *Streptococcus oralis*, and *Candida albicans* formed on four types of clear aligner materials. The results clearly show that biofilm inhibition depends on both the chemical composition of the mouthwash and the physical–chemical properties of the aligner material, confirming the multifactorial nature of biofilm control in clear aligner orthodontic therapy.

Across all microorganisms and materials, MW-D (chlorhexidine-based with fluoride) showed the most consistent efficacy, delivering statistically superior inhibition against bacterial and fungal biofilms. MW-B (fluoride with cetylpyridinium chloride) also performed strongly, especially for *S. oralis* and *C. albicans*, often matching or closely trailing MW-D. MW-G (essential oils with xylitol) was particularly effective against fungal *C. albicans*, confirming previous findings on antifungal activity of essential oils [22,23]. MW-E (plant-based, low fluoride) and MW-F (aminfluoride-based) demonstrated the lowest inhibition values.

Occasional negative BIP% values were observed for *C. albicans* following exposure to the plant-based formulation MW-E, which contains peppermint oil, aloe vera, green tea, and chamomile extracts along with xylitol, sorbitol, and low fluoride. These results likely reflect metabolic stimulation by nutritive or antioxidant constituents capable of supporting limited microbial activity, coupled with strain-specific boosting effects in which subinhibitory phytochemical or fluoride exposure transiently enhances cell metabolism or adhesion. Such near-zero or slightly negative inhibition percentages (≤5%) are biologically plausible and fall within the expected range of variability for natural formulations, without influencing the overall comparative interpretation of mouthwash efficacy.

Material composition significantly influenced outcomes. The monolayer polyurethane thermoformed material (Material B) consistently yielded the highest inhibition rates, particularly against *S. oralis* and *C. albicans*. The modified monolayer polyurethane with increased modulus (Material A) was most effective for *S. mutans* and performed competitively overall. In contrast, Materials C and D (multilayer and copolyester-based, respectively) showed lower inhibition, likely due to differences in surface free energy and roughness influencing microbial adhesion [24,25,26].

Organism-specific patterns emerged. *S. mutans* was most susceptible to chlorhexidine-based and alcohol-containing essential oil formulations—consistent with their broad antibacterial action [27,28,29]. *S. oralis* was particularly sensitive to CPC and chlorhexidine rinse formulations, reflecting known species-specific susceptibility to cationic antiseptics [30]. *C. albicans* showed strong inhibition with MW-D and MW-G, aligning with established antifungal mechanisms via essential oils or chlorhexidine-induced membrane disruption [31,32]. Occasional negative inhibition values in some formulations might indicate nutrient effects from botanical extracts or insufficient antifungal action.

A key strength of this study is the comprehensive standardized assessment of three clinically relevant oral microorganisms—two bacterial and one fungal—on multiple aligner materials under uniform conditions and a clinically relevant one-minute contact time, as recommended by professional guidelines [19]. The optical density–based quantification method allowed for precise intergroup comparisons and statistical validation.

Clear aligner therapy presents unique challenges for biofilm management due to prolonged intraoral wear, intimate adaptation to tooth surfaces, and frequent removal–reinsertion cycles, which may facilitate repeated microbial colonization unless proper cleaning is performed before each reinsertion [33]. The present findings suggest that not all aligner materials respond equally to chemical biofilm control, which may be related to differences in polymer composition, surface hydrophobicity, and elastic recovery after deformation. The differences in biofilm inhibition observed among the four aligner materials can be further interpreted in the context of their intrinsic surface characteristics. Polyurethane-based materials (Materials A and B) generally exhibit smoother topography, lower surface roughness, and higher flexibility compared with copolyester-based systems (Material D), which tend to present greater micro-roughness and surface energy variations [24,25,26,34,35]. These parameters directly influence microbial adhesion and subsequent biofilm maturation by modulating the interaction forces between microbial cells and the polymer surface. Smoother, more hydrophobic polyurethane surfaces are known to reduce initial bacterial anchoring, whereas copolyester substrates may promote more stable microcolonies due to their higher wettability and surface heterogeneity. The superior inhibition values recorded for the monolayer polyurethane aligners in this study are consistent with these surface-dependent adhesion mechanisms, suggesting that material composition and topography significantly modulate the efficacy of chemical biofilm control strategies. In particular, the superior performance of the monolayer polyurethane thermoformed material (Material B) and the modified monolayer polyurethane (Material A) could be attributed to their smoother surface topography and higher hydrophobicity relative to multilayer or copolyester-based materials. Although direct surface characterization was not performed in this study, previous investigations have shown that polyurethane aligner systems typically exhibit lower surface roughness and greater hydrophobicity than copolyester or multilayer formulations, properties that can influence microbial adhesion and biofilm resilience [24,35].

These results underscore the potential value of integrating material-specific biofilm susceptibility profiles into oral hygiene recommendations for patients undergoing clear aligner treatment, thereby optimizing microbial control and reducing the risk of caries, periodontal inflammation, and opportunistic infections such as candidiasis.

The distinct antimicrobial mechanisms of the tested mouthwashes may also explain the organism- and material-specific responses observed. Chlorhexidine digluconate exerts its effect by disrupting bacterial cell membranes and causing cytoplasmic leakage, resulting in broad-spectrum inhibition of *Streptococcus* species and *Candida albicans* even at low concentrations [36]. Cetylpyridinium chloride (CPC), a quaternary ammonium compound, provides rapid surface activity by binding to negatively charged microbial membranes and inducing cell lysis, though its efficacy can be modulated by surface adsorption and organic load [37]. In contrast, essential-oil formulations containing phenolic and terpenic compounds act primarily by altering cell wall permeability, inhibiting enzymatic activity, and disrupting quorum-sensing pathways [38,39]. The variable inhibition patterns recorded among the polyurethane- and copolyester-based aligners suggest that polymer hydrophobicity and surface energy modulate the interaction between active molecules and adherent biofilm cells, influencing diffusion and retention of antiseptic agents on the aligner surface.

While the present in vitro study focused on a single short-term contact period to allow controlled comparisons among products, this simplified design does not fully reproduce the cyclic exposure and salivary conditioning that occur intraorally. Future investigations should therefore incorporate salivary pellicle deposition, intermittent rinsing cycles, and time-kill kinetic assays to more accurately represent the dynamic antimicrobial environment encountered during daily aligner wear.

As no chemical neutralizer was applied after mouthwash exposure, a slight residual antimicrobial effect cannot be fully excluded; however, since all samples were treated identically, any potential bias is expected to be minimal, systematic across groups, and likely to result in a modest overestimation of inhibition rather than altering comparative trends. Limitations of these findings include the in vitro nature of the study, absence of mixed-species biofilms, and evaluation of only single exposure and incubation times. Future research should explore repeated exposures, diverse biofilm communities, and in vivo validation to enhance the clinical applicability of findings.

## 5. Conclusions

The present findings demonstrate that both mouthwash formulation and aligner material composition markedly influence biofilm inhibition against *Streptococcus mutans, Streptococcus oralis*, and *Candida albicans.* The chlorhexidine-based rinse (MW-D) exhibited the most consistent and effective antibacterial and antifungal activity, while the fluoride-cetylpyridinium chloride formulation (MW-B) proved to be a strong alternative in specific microbial contexts. Among the tested aligner materials, the monolayer polyurethane thermoformed material (Material B) achieved the highest overall inhibition rates, with the modified monolayer polyurethane (Material A) showing comparably strong results in several instances.

These results highlight the importance of selecting appropriate adjunctive hygiene products in relation to the aligner material used, supporting a more tailored approach to biofilm control in clear aligner therapy. Future research should explore multispecies biofilms, repeated rinsing protocols, and in vivo validation to expand the clinical applicability of these findings.

## Figures and Tables

**Table 1:** Tested Mouthwash Solutions with Descriptive Characteristics and Key Active Ingredients.

Mouthwash Code	Descriptive Characteristics	Key Active Ingredients
MW-A	Essential oil-based mouthwash with fluoride	Mint (*Mentha arvensis*) essential oil; sage (*Salvia officinalis*) extract; aloe vera leaf extract; sodium fluoride (~905 ppm F^−^)
MW-B	Fluoride-containing antiseptic mouthwash	Sodium fluoride (~225 ppm F^−^), cetylpyridinium chloride (0.05%), menthol
MW-C	Alcohol-based essential oil fluoride mouthwash	Eucalyptol, thymol, menthol, methyl salicylate; zinc chloride (~0.09%); sodium fluoride (~220 ppm F^−^); ethanol (~21% *v*/*v*)
MW-D	Chlorhexidine-based antiseptic fluoride rinse (no alcohol)	Chlorhexidine digluconate 0.05%, sodium fluoride (~250 ppm F^−^), herb extracts (chamomile, *Krameria triandra*, *Commiphora myrrha*, *Potentilla erecta*, *Salvia officinalis*), bisabolol
MW-E	Plant-based natural mouthwash (vegan, alcohol-free, low-fluoride)	Organic peppermint oil, aloe vera leaf extract, green tea (*Camellia sinensis*) extract, chamomile (*Chamomilla recutita*) extract; sodium fluoride (~226 ppm F^−^), xylitol, sorbitol
MW-F	Amin-fluoride-based (sensitivity-targeted), alcohol-free	Amine fluoride (Olaflur ~250 ppm F^−^), sodium fluoride, arginine (Pro-Argin technology), menthol, glycerin, PEG-40 castor oil, propylene glycol, sodium saccharin
MW-G	Essential oil-based natural mouthwash with xylitol (no alcohol)	Wild orange (*Citrus sinensis*) peel oil, clove bud (*Syzygium aromaticum*) oil, cinnamon bark oil, eucalyptus (*Eucalyptus globulus*) oil, rosemary (*Rosmarinus officinalis*) oil; xylitol, water, glycerin, sodium bicarbonate

## Data Availability

The data presented in this study are available on request from the corresponding author. Commercial brand names of mouthwashes and aligners have been anonymized in the publicly available data to avoid potential commercial bias or conflicts of interest.

## Supplementary Materials

The following supporting information can be downloaded at: https://www.mdpi.com/article/10.3390/jfb16110424/s1; Table S1: Data concerning OD_540_ measurements, mean ± SD, 95% confidence intervals, and calculated bacterial/fungal growth (BGP/FGP %) and bacterial/fungal inhibition (BIP/FIP %) values.
