# Supplementary material for: In Vitro Evaluation of Biofilm Formation by Oral Microorganisms on Clear Aligner Materials: Influence of Mouthwash Exposure
Source: J Funct Biomater. 2025 Nov 13;16(11):424. doi: 10.3390/jfb16110424 (PMC12653573; doi:10.3390/jfb16110424)
Supplement: Supplementary file 1 [file jfb-16-00424-s001.zip › jfb-3936888-supplementary.pdf]

**Supplemental Table S1 –** Data concerning $OD_{540}$ measurements, mean ± SD, 95 % confidence intervals, and calculated bacterial growth (BGP %) and bacterial inhibition (BIP %) values

a) *S. mutans*

| | MW-A | MW-B | MW-C | MW-D | MW-E | MW-F | MW-G | Material |
|---|---|---|---|---|---|---|---|---|
| Rep 1 | 0.604 | 0.580 | 0.515 | 0.774 | 0.921 | 0.725 | 0.673 | A |
| Rep 2 | 0.660 | 0.568 | 0.515 | 0.882 | 0.936 | 0.844 | 0.610 | |
| Rep 3 | 0.584 | 0.529 | 0.605 | 0.843 | 0.861 | 0.786 | 0.634 | |
| **Mean** | **0.616** | **0.559** | **0.545** | **0.833** | **0.906** | **0.785** | **0.639** | |
| SD | 0.040 | 0.026 | 0.052 | 0.055 | 0.040 | 0.060 | 0.032 | |
| 95 % CI low | 0.517 | 0.494 | 0.416 | 0.697 | 0.808 | 0.637 | 0.560 | |
| 95 % CI low | 0.715 | 0.624 | 0.674 | 0.969 | 1.004 | 0.933 | 0.718 | |
| BGP% | 62.54 | 56.75 | 55.33 | 84.57 | 91.98 | 79.70 | 64.87 | |
| BIP% | 37.46 | 43.25 | 44.67 | 15.43 | 8.02 | 20.30 | 35.13 | |
| Rep 1 | 0.652 | 0.890 | 0.658 | 0.959 | 0.889 | 0.931 | 0.849 | B |
| Rep 2 | 0.626 | 0.787 | 0.699 | 0.862 | 0.927 | 0.938 | 0.874 | |
| Rep 3 | 0.543 | 0.795 | 0.662 | 0.844 | 1.016 | 1.006 | 0.741 | |
| **Mean** | **0.607** | **0.824** | **0.673** | **0.888** | **0.944** | **0.958** | **0.821** | |
| SD | 0.057 | 0.057 | 0.022 | 0.062 | 0.065 | 0.042 | 0.070 | |
| 95 % CI low | 0.466 | 0.682 | 0.617 | 0.735 | 0.781 | 0.854 | 0.646 | |
| 95 % CI low | 0.748 | 0.966 | 0.729 | 1.041 | 1.107 | 1.062 | 0.996 | |
| BGP% | 61.62 | 83.65 | 68.32 | 90.15 | 95.84 | 97.26 | 83.35 | |
| BIP% | 38.38 | 16.35 | 31.68 | 9.85 | 4.16 | 2.74 | 16.65 | |
| Rep 1 | 0.5649 | 0.5455 | 0.82 | 0.7204 | 0.9787 | 0.9027 | 0.849 | C |
| Rep 2 | 0.621 | 0.5701 | 0.9486 | 0.6265 | 0.8591 | 0.9509 | 0.874 | |
| Rep 3 | 0.6081 | 0.5854 | 0.8985 | 0.6841 | 0.8983 | 0.9515 | 0.741 | |
| **Mean** | **0.598** | **0.567** | **0.889** | **0.677** | **0.912** | **0.935** | **0.821** | |
| SD | 0.029 | 0.020 | 0.065 | 0.047 | 0.061 | 0.028 | 0.070 | |
| 95 % CI low | 0.525 | 0.517 | 0.728 | 0.559 | 0.761 | 0.865 | 0.646 | |
| 95 % CI low | 0.671 | 0.617 | 1.050 | 0.795 | 1.063 | 1.005 | 0.996 | |
| BGP% | 60.71 | 57.56 | 90.25 | 68.73 | 92.59 | 94.92 | 76.45 | |
| BIP% | 39.29 | 42.44 | 9.75 | 31.27 | 7.41 | 5.08 | 23.55 | |
| Rep 1 | 0.6917 | 0.9339 | 0.8979 | 0.761 | 1.021 | 0.9812 | 0.849 | D |
| Rep 2 | 0.7352 | 0.953 | 0.9852 | 0.6827 | 1.0087 | 0.9769 | 0.874 | |
| Rep 3 | 0.6731 | 0.8131 | 0.8619 | 0.7042 | 0.9193 | 0.8139 | 0.741 | |
| **Mean** | **0.700** | **0.900** | **0.915** | **0.716** | **0.983** | **0.924** | **0.821** | |

| | | | | | | | | |
|---|---|---|---|---|---|---|---|---|
| SD | 0.032 | 0.076 | 0.063 | 0.040 | 0.056 | 0.095 | 0.070 | |
| 95 % CI low | 0.621 | 0.712 | 0.757 | 0.615 | 0.845 | 0.687 | 0.646 | |
| 95 % CI low | 0.779 | 1.088 | 1.073 | 0.816 | 1.121 | 1.161 | 0.996 | |
| BGP% | 71.07 | 91.37 | 92.89 | 72.69 | 99.80 | 93.81 | 60.91 | |
| BIP% | 28.93 | 8.63 | 7.11 | 27.31 | 0.20 | 6.19 | 39.09 | |

b) *S. oralis*

| | MW-A | MW-B | MW-C | MW-D | MW-E | MW-F | MW-G | Materia l |
|---|---|---|---|---|---|---|---|---|
| Rep 1 | 0.562 | 0.191 | 0.589 | 0.667 | 0.806 | 1.007 | 0.673 | A |
| Rep 2 | 0.525 | 0.185 | 0.591 | 0.680 | 0.877 | 0.976 | 0.610 | |
| Rep 3 | 0.476 | 0.218 | 0.602 | 0.810 | 0.894 | 0.876 | 0.634 | |
| **Mean** | **0.521** | **0.198** | **0.594** | **0.719** | **0.859** | **0.953** | **0.639** | |
| SD | 0.043 | 0.018 | 0.007 | 0.079 | 0.047 | 0.069 | 0.032 | |
| 95 % CI low | 0.414 | 0.154 | 0.577 | 0.523 | 0.743 | 0.782 | 0.560 | |
| 95 % CI low | 0.628 | 0.242 | 0.611 | 0.915 | 0.975 | 1.124 | 0.718 | |
| BGP% | 52.89 | 20.10 | 60.30 | 72.99 | 87.21 | 96.75 | 29.85 | |
| BIP% | 47.11 | 79.90 | 39.70 | 27.01 | 12.79 | 3.25 | 70.15 | |
| Rep 1 | 0.5027 | 0.155 | 0.8091 | 0.1692 | 0.9454 | 0.7896 | 0.2272 | B |
| Rep 2 | 0.4534 | 0.1627 | 0.8982 | 0.1758 | 0.9302 | 0.7954 | 0.2131 | |
| Rep 3 | 0.5109 | 0.1593 | 0.8067 | 0.192 | 0.9204 | 0.914 | 0.2257 | |
| **Mean** | **0.489** | **0.159** | **0.838** | **0.179** | **0.932** | **0.833** | **0.222** | |
| SD | 0.031 | 0.004 | 0.052 | 0.012 | 0.013 | 0.070 | 0.008 | |
| 95 % CI low | 0.412 | 0.149 | 0.708 | 0.150 | 0.901 | 0.659 | 0.203 | |
| 95 % CI low | 0.566 | 0.169 | 0.968 | 0.208 | 0.963 | 1.007 | 0.241 | |
| BGP% | 49.64 | 16.14 | 85.08 | 18.17 | 94.62 | 84.57 | 22.54 | |
| BIP% | 50.36 | 83.86 | 14.92 | 81.83 | 5.38 | 15.43 | 77.46 | |
| Rep 1 | 0.7963 | 0.1803 | 0.9143 | 0.1815 | 0.9135 | 0.8077 | 0.7002 | C |
| Rep 2 | 0.7184 | 0.17 | 0.7834 | 0.1735 | 0.919 | 0.8552 | 0.655 | |
| Rep 3 | 0.7563 | 0.1597 | 0.8553 | 0.155 | 0.9965 | 0.908 | 0.6247 | |
| **Mean** | **0.757** | **0.170** | **0.851** | **0.170** | **0.943** | **0.857** | **0.660** | |
| SD | 0.039 | 0.010 | 0.066 | 0.014 | 0.046 | 0.050 | 0.038 | |
| 95 % CI low | 0.660 | 0.144 | 0.688 | 0.136 | 0.828 | 0.732 | 0.566 | |
| 95 % CI low | 0.854 | 0.196 | 1.014 | 0.204 | 1.058 | 0.982 | 0.754 | |

| | | | | | | | | |
|---|---|---|---|---|---|---|---|---|
| BGP% | 76.85 | 17.26 | 86.40 | 17.26 | 95.74 | 87.01 | 67.01 | |
| BIP% | 23.15 | 82.74 | 13.60 | 82.74 | 4.26 | 12.99 | 32.99 | |
| Rep 1 | 0.5527 | 0.1843 | 0.8245 | 0.9963 | 0.9476 | 0.8278 | 0.6368 | D |
| Rep 2 | 0.5714 | 0.1666 | 0.8087 | 1.0383 | 0.8597 | 0.8815 | 0.6909 | |
| Rep 3 | 0.6218 | 0.1741 | 0.6858 | 0.8514 | 0.8866 | 0.8467 | 0.7123 | |
| **Mean** | **0.582** | **0.175** | **0.773** | **0.962** | **0.898** | **0.852** | **0.680** | |
| SD | 0.036 | 0.009 | 0.076 | 0.098 | 0.045 | 0.027 | 0.039 | |
| 95 % CI low | 0.493 | 0.153 | 0.584 | 0.718 | 0.786 | 0.784 | 0.583 | |
| 95 % CI low | 0.671 | 0.197 | 0.962 | 1.206 | 1.010 | 0.920 | 0.777 | |
| BGP% | 59.09 | 17.77 | 78.48 | 97.66 | 91.17 | 86.50 | 69.04 | |
| BIP% | 40.91 | 82.23 | 21.52 | 2.34 | 8.83 | 13.50 | 30.96 | |

c) *C. albicans*

| | MW-A | MW-B | MW-C | MW-D | MW-E | MW-F | MW-G | Material |
|---|---|---|---|---|---|---|---|---|
| Rep 1 | 0.7132 | 0.7681 | 0.6424 | 0.2264 | 0.8894 | 0.931 | 0.2466 | A |
| Rep 2 | 0.7285 | 0.7423 | 0.6838 | 0.2212 | 0.8469 | 0.8045 | 0.2452 | |
| Rep 3 | 0.8053 | 0.8715 | 0.6538 | 0.2154 | 0.7537 | 0.8536 | 0.2672 | |
| **Mean** | **0.749** | **0.794** | **0.660** | **0.221** | **0.830** | **0.863** | **0.253** | |
| SD | 0.049 | 0.068 | 0.021 | 0.006 | 0.069 | 0.064 | 0.012 | |
| 95 % CI low | 0.626 | 0.624 | 0.607 | 0.207 | 0.658 | 0.705 | 0.222 | |
| 95 % CI low | 0.872 | 0.964 | 0.713 | 0.235 | 1.002 | 1.021 | 0.284 | |
| BGP% | 78.51 | 84.92 | 70.59 | 23.64 | 88.77 | 92.30 | 27.06 | |
| BIP% | 21.49 | 15.08 | 29.41 | 76.36 | 11.23 | 7.70 | 72.94 | |
| Rep 1 | 0.8853 | 0.2145 | 0.9339 | 0.1764 | 0.9702 | 0.576 | 0.6065 | B |
| Rep 2 | 0.893 | 0.2198 | 0.9555 | 0.1935 | 0.8905 | 0.5575 | 0.6682 | |
| Rep 3 | 0.8707 | 0.2617 | 0.8317 | 0.1762 | 0.9503 | 0.5796 | 0.6423 | |
| **Mean** | **0.883** | **0.232** | **0.907** | **0.182** | **0.937** | **0.571** | **0.639** | |
| SD | 0.011 | 0.026 | 0.066 | 0.010 | 0.041 | 0.012 | 0.031 | |
| 95 % CI low | 0.855 | 0.168 | 0.743 | 0.157 | 0.834 | 0.542 | 0.562 | |
| 95 % CI low | 0.911 | 0.296 | 1.071 | 0.207 | 1.040 | 0.600 | 0.716 | |
| BGP% | 92.56 | 24.32 | 95.07 | 19.08 | 98.22 | 59.85 | 66.98 | |
| BIP% | 7.44 | 75.68 | 4.93 | 80.92 | 1.78 | 40.15 | 33.02 | |
| Rep 1 | 0.8846 | 0.1732 | 0.8676 | 0.4415 | 0.9419 | 0.9696 | 0.2687 | C |
| Rep 2 | 0.823 | 0.1825 | 0.8466 | 0.4719 | 0.9575 | 0.8613 | 0.2958 | |
| Rep 3 | 0.8994 | 0.1693 | 0.9767 | 0.5206 | 1.0496 | 0.8661 | 0.2995 | |

| | | | | | | | | |
|---|---|---|---|---|---|---|---|---|
| **Mean** | **0.869** | **0.175** | **0.897** | **0.478** | **0.983** | **0.899** | **0.288** | |
| SD | 0.041 | 0.007 | 0.070 | 0.040 | 0.058 | 0.061 | 0.017 | |
| 95 % CI low | 0.768 | 0.158 | 0.723 | 0.379 | 0.838 | 0.747 | 0.246 | |
| 95 % CI low | 0.970 | 0.192 | 1.070 | 0.577 | 1.128 | 1.051 | 0.330 | |
| BGP% | 91.09 | 18.34 | 94.03 | 50.10 | 103.04 | 94.23 | 30.19 | |
| BIP% | 8.91 | 81.66 | 5.97 | 49.90 | -3.04 | 5.77 | 69.81 | |
| Rep 1 | 0.7118 | 0.7017 | 0.8286 | 0.796 | 0.9396 | 0.8751 | 0.5805 | D |
| Rep 2 | 0.7239 | 0.6922 | 0.7754 | 0.82 | 1.0524 | 0.8147 | 0.665 | |
| Rep 3 | 0.8413 | 0.8051 | 0.757 | 0.751 | 0.945 | 0.7582 | 0.6415 | |
| **Mean** | **0.759** | **0.733** | **0.787** | **0.789** | **0.979** | **0.816** | **0.629** | |
| SD | 0.072 | 0.063 | 0.037 | 0.035 | 0.064 | 0.058 | 0.044 | |
| 95 % CI low | 0.581 | 0.577 | 0.695 | 0.702 | 0.821 | 0.671 | 0.521 | |
| 95 % CI low | 0.937 | 0.889 | 0.879 | 0.876 | 1.137 | 0.961 | 0.737 | |
| BGP% | 79.56 | 76.83 | 82.49 | 82.70 | 102.62 | 85.53 | 65.93 | |
| BIP% | 20.44 | 23.17 | 17.51 | 17.30 | -2.62 | 14.47 | 34.07 | |
